# Global Entrainment of Transcriptional Systems to Periodic Inputs

**DOI:** 10.1371/journal.pcbi.1000739

**Published:** 2010-04-15

**Authors:** Giovanni Russo, Mario di Bernardo, Eduardo D. Sontag

**Affiliations:** 1 Department of Systems and Computer Engineering, University of Naples Federico II, Naples, Italy; 2 Department of Engineering Mathematics, University of Bristol, Bristol, United Kingdom; 3 Department of Mathematics, Rutgers University, Piscataway, New Jersey, United States; University of Washington, United States of America

## Abstract

This paper addresses the problem of providing mathematical conditions that allow one to ensure that biological networks, such as transcriptional systems, can be globally entrained to external periodic inputs. Despite appearing obvious at first, this is by no means a generic property of nonlinear dynamical systems. Through the use of contraction theory, a powerful tool from dynamical systems theory, it is shown that certain systems driven by external periodic signals have the property that all their solutions converge to a fixed limit cycle. General results are proved, and the properties are verified in the specific cases of models of transcriptional systems as well as constructs of interest in synthetic biology. A self-contained exposition of all needed results is given in the paper.

## Author Summary

The activities of living organisms are governed by complex sets of biochemical reactions. Often, entrainment to certain external signals helps control the timing and sequencing of reactions. An important open problem is to understand the onset of entrainment and under what conditions it can be ensured in the presence of uncertainties, noise, and environmental variations. In this paper, we focus mainly on transcriptional systems, modeled by Ordinary Differential Equations. These are basic building blocks for more complex biochemical systems. However, the results that we obtain are of more generality. To illustrate this generality, and to emphasize the use of our techniques in synthetic biology, we discuss the entrainment of a Repressilator circuit and the synchronization of a network of Repressilators. We answer the following two questions: 1) What are the dynamical mechanisms that ensure the entrainment to periodic inputs in transcriptional modules? 2) Starting from natural systems, what properties can be used to design novel synthetic biological circuits that can be entrained? For some biological systems which are always “in contact” with a continuously changing environment, entrainment may be a “desired” property. Thus, answering the above two questions is of fundamental importance. While entrainment may appear obvious at first thought, it is not a generic property of nonlinear dynamical systems. The main result of our paper shows that, even if the transcriptional modules are modeled by nonlinear ODEs, they can be entrained by any (positive) periodic signal. Surprisingly, such a property is preserved if the system parameters are varied: entrainment is obtained independently of the particular biochemical conditions. We prove that combinations of the above transcriptional module also show the same property. Finally, we show how the developed tools can be applied to design synthetic biochemical systems guaranteed to exhibit entrainment.

## Introduction

Periodic, clock-like rhythms pervade nature and regulate the function of all living organisms. For instance, *circadian rhythms* are regulated by an endogenous biological clock entrained by the light signals from the environment that then acts as a pacemaker [1]. Moreover, such an entrainment can be obtained even if daily variations are present, like e.g. temperature and light variations. Another important example of entrainment in biological systems is at the molecular level, where the synchronization of several cellular processes is regulated by the cell cycle [2].

An important question in mathematical and computational biology is that of finding conditions ensuring that entrainment occurs. The objective is to identify classes of biological systems that can be entrained by an exogenous signal. To solve this problem, modelers often resort to simulations in order to show the existence of periodic solutions in the system of interest. Simulations, however, can never prove that solutions will exist for all parameter values, and they are subject to numerical errors. Moreover, robustness of entrained solutions needs to be checked in the presence of noise and uncertainties, which cannot be avoided experimentally.

From a mathematical viewpoint, the problem of formally showing that entrainment takes place is known to be extremely difficult. Indeed, if a stable linear time-invariant model is used to represent the system of interest, then entrainment is usually expected, when the system is driven by an external periodic input, with the system response being a filtered, shifted version of the external driving signal. However, in general, as is often the case in biology, models are nonlinear. The response of nonlinear systems to periodic inputs is the subject of much current systems biology experimentation; for example, in [3], the case of a cell signaling system driven by a periodic square-wave input is considered. From measurements of a periodic output, the authors fit a transfer function to the system, implicitly modeling the system as linear even though (as stated in the Supplemental Materials to [3]) there are saturation effects so the true system is nonlinear. For nonlinear systems, driving the system by an external periodic signal does not guarantee the system response to also be a periodic solution, as nonlinear systems can exhibit harmonic generation or suppression and complex behavior such as chaos or quasi-periodic solutions [4]. This may happen even if the system is well-behaved with respect to constant inputs; for example, there are systems which converge to a fixed steady state no matter what is the input excitation, so long as this input signal is constant, yet respond chaotically to the simplest oscillatory input; we outline such an example in the Materials and Methods Section, see also [5]. Thus, a most interesting open problem is that of finding conditions for the entrainment to external inputs of biological systems modeled by sets of nonlinear differential equations.

One approach to analyzing the convergence behavior of nonlinear dynamical systems is to use Lyapunov functions. However, in biological applications, the appropriate Lyapunov functions are not always easy to find and, moreover, convergence is not guaranteed in general in the presence of noise and/or uncertainties. Also, such an approach can be hard to apply to the case of non-autonomous systems (that is, dynamical systems directly dependent on time), as is the case when dealing with periodically forced systems.

The above limitations can be overcome if the convergence problem is interpreted as a property of all trajectories, asking that all solutions converge towards one another (contraction). This is the viewpoint of *contraction theory*, [6], [7], and more generally incremental stability methods [8]. Global results are possible, and these are robust to noise, in the sense that, if a system satisfies a contraction property then trajectories remain bounded in the phase space [9]. Contraction theory has a long history. Contractions in metric functional spaces can be traced back to the work of Banach and Caccioppoli [10] and, in the field of dynamical systems, to [11] and even to [12] (see also [13], [8], and e.g. [14] for a more exhaustive list of related references). Contraction theory has been successfully applied to both nonlinear control and observer problems, [7], [15] and, more recently, to synchronization and consensus problems in complex networks [16], [17],[18]. In [19] it was proposed that contraction can be particularly useful when dealing with the analysis and characterization of biological networks. In particular, it was found that using non Euclidean norms, as also suggested in [6] (Sec. 3.7ii), can be particularly effective in this context [19], [20].

One of the objectives of this paper is to give a self-contained exposition, with all proofs included, of results in contraction theory as applied to entrainment of periodic signals, and, moreover, to show their applicability to problems of biological interest. *We believe that contraction analysis should be recognized as an important component of the “toolkit” of systems biology*, and this paper should be useful to other researchers contemplating the use of these tools.

For concreteness, we focus mainly on transcriptional systems, as well as related biochemical systems, which are basic building blocks for more complex biochemical systems. However, the results that we obtain are of more generality. To illustrate this generality, and to emphasize the use of our techniques in synthetic biology design, we discuss as well the entrainment of a Repressilator circuit in a parameter regime in which endogenous oscillations do not occur, as well as the synchronization of a network of Repressilators. A surprising fact is that, for these applications, and contrary to many engineering applications, norms other than Euclidean, and associated matrix measures, must be considered.

### Mathematical tools

We consider in this paper systems of ordinary differential equations, generally time-dependent:
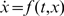
(1)defined for 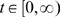 and 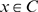, where 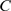 is a subset of 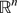. It will be assumed that 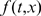 is differentiable on 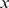, and that 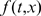, as well as the Jacobian of 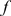 with respect to 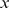, denoted as 
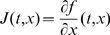
, are both continuous in 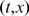. In applications of the theory, it is often the case that 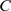 will be a closed set, for example given by non-negativity constraints on variables as well as linear equalities representing mass-conservation laws. For a non-open set 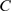, differentiability in 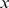 means that the vector field 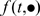 can be extended as a differentiable function to some open set which includes 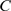, and the continuity hypotheses with respect to 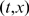 hold on this open set.

We denote by 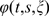 the value of the solution 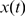 at time 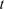 of the differential equation (1) with initial value 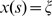. It is implicit in the notation that 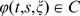 (“forward invariance” of the state set 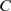). This solution is in principle defined only on some interval 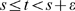, but we will assume that 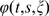 is defined for all 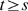. Conditions which guarantee such a “forward-completeness” property are often satisfied in biological applications, for example whenever the set 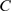 is closed and bounded, or whenever the vector field 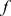 is bounded. (See Appendix C in [21] for more discussion, as well as [22] for a characterization of the forward completeness property.) Under the stated assumptions, the function 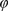 is jointly differentiable in all its arguments (this is a standard fact on well-posedness of differential equations, see for example Appendix C in [21]).

We recall (see for instance [23]) that, given a vector norm on Euclidean space (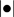), with its induced matrix norm 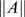, the associated *matrix measure* 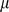 is defined as the directional derivative of the matrix norm, that is,
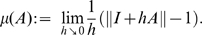
For example, if 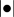 is the standard Euclidean 2-norm, then 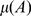 is the maximum eigenvalue of the symmetric part of 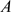. As we shall see, however, different norms will be useful for our applications. Matrix measures are also known as “*logarithmic norms*”, a concept independently introduced by Germund Dahlquist and Sergei Lozinskii in 1959, [24],[25]. The limit is known to exist, and the convergence is monotonic, see [24], [26].

We will say that system (1) is *infinitesimally contracting* on a convex set 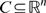 if there exists some norm in 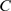, with associated matrix measure 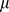 such that, for some constant 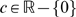,
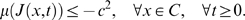
(2)


Let us discuss informally (rigorous proofs are given later) the motivation for this concept. Since by assumption 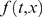 is continuously differentiable, the following exact *differential* relation can be obtained from (1):
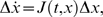
(3)where, as before, 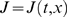 denotes the Jacobian of the vector field 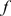, as a function of 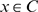 and 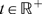, and where 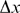 denotes a small change in states and “
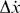
” means 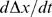, evaluated along a trajectory. (In mechanics, as in [27], 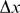 is called “virtual displacement”, and formally it may be thought of as a linear tangent differential form, differentiable with respect to time.) Consider now two neighboring trajectories of (1), evolving in 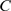, and the virtual displacements between them. Note that (3) can be thought of as a linear time-varying dynamical system of the form:
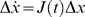
once that 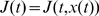 is thought of as a fixed function of time. Hence, an upper bound for the magnitude of its solutions can be obtained by means of the Coppel inequality [28], yielding:
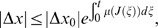
(4)where 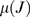 is the matrix measure of the system Jacobian induced by the norm being considered on the states and 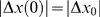. Using (4) and (2), we have that
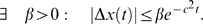
Thus, trajectories starting from infinitesimally close initial conditions converge exponentially towards each other. In what follows we will refer to 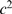 as *contraction (or convergence) rate*.

The key theoretical result about contracting systems links infinitesimal and global contractivity, and is stated below. This result can be traced, under different technical assumptions, to e.g. [6], [13], [12], [11].

**Theorem 1.** *Suppose that* 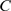 *is a convex subset of* 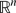 *and that* 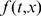 *is infinitesimally contracting with contraction rate* 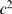 *. Then, for every two solutions* 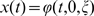 *and* 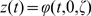 *of (1), it holds that:*

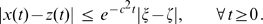
(5)


In other words, infinitesimal contractivity implies global contractivity. In the Materials and Methods section, we provide a self-contained proof of Theorem 1. In fact, the result is shown there in a generalized form, in which convexity is replaced by a weaker constraint on the geometry of the space.

In actual applications, often one is given a system which depends implicitly on the time, 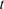, by means of a continuous function 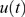, i.e. systems dynamics are represented by 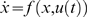. In this case, 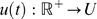 (where 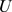 is some subset of 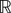), represents an external input. It is important to observe that the contractivity property does not require any prior information about this external input. In fact, since 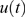 does not depend on the system state variables, when checking the property, it may be viewed as a constant parameter, 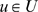. Thus, if contractivity of 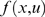 holds uniformly 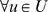, then it will also hold for 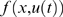.

Given a number 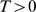, we will say that system (1) is 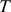 *-periodic* if it holds that
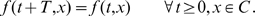
Notice that the system 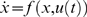 is 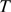-periodic, if the external input, 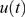, is itself a periodic function of period 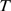.

The following is the basic theoretical result about periodic orbits that will be used in the paper. A proof may be found in [6], Sec. 3.7.vi.

**Theorem 2.** *Suppose that:*

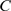
 *is a closed convex subset of* 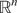;

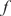
 *is infinitesimally contracting with contraction rate* 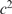;

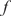
 *is* 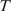 *-periodic.*

*Then, there is a unique periodic solution* 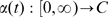 *of (1) of period* 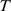 *and, for every solution* 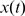 *, it holds that* 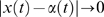 *as* 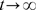.

In the Materials and Methods section of this paper, we provide a self-contained proof of Theorem 2, in a generalized form which does not require convexity.

### A simple example

As a first example to illustrate the application of the concepts introduced so far, we choose a simple bimolecular reaction, in which a molecule of 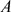 and one of 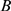 can reversibly combine to produce a molecule of 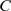.

This system can be modeled by the following set of differential equations:
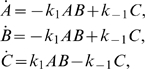
(6)where we are using 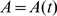 to denote the concentration of 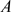 and so forth. The system evolves in the positive orthant of 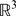. Solutions satisfy (stoichiometry) constraints:
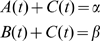
(7)for some constants 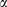 and 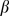.

We will assume that one or both of the “kinetic constants” 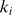 are time-varying, with period 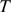. Such a situation arises when the 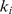
's depend on concentrations of additional enzymes, which are available in large amounts compared to the concentrations of 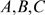, but whose concentrations are periodically varying. The only assumption will be that 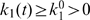 and 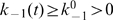 for all 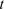.

Because of the conservation laws (7), we may restrict our study to the equation for 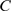. Once that all solutions of this equation are shown to globally converge to a periodic orbit, the same will follow for 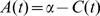 and 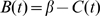. We have that:
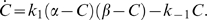
(8)Because 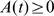 and 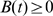, this system is studied on the subset of 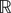 defined by 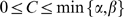. The equation can be rewritten as:
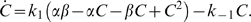
(9)Differentiation with respect to 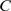 of the right-hand side in the above system yields this (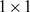) Jacobian:
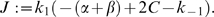
(10)Since we know that 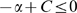 and 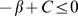, it follows that
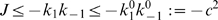
for 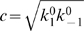. Using any norm (this example is in dimension one) we have that 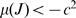. So (6) is contracting and, by means of Theorem 2, solutions will globally converge to a unique solution of period 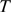 (notice that such a solution depends on system parameters).

**Figure 1:**
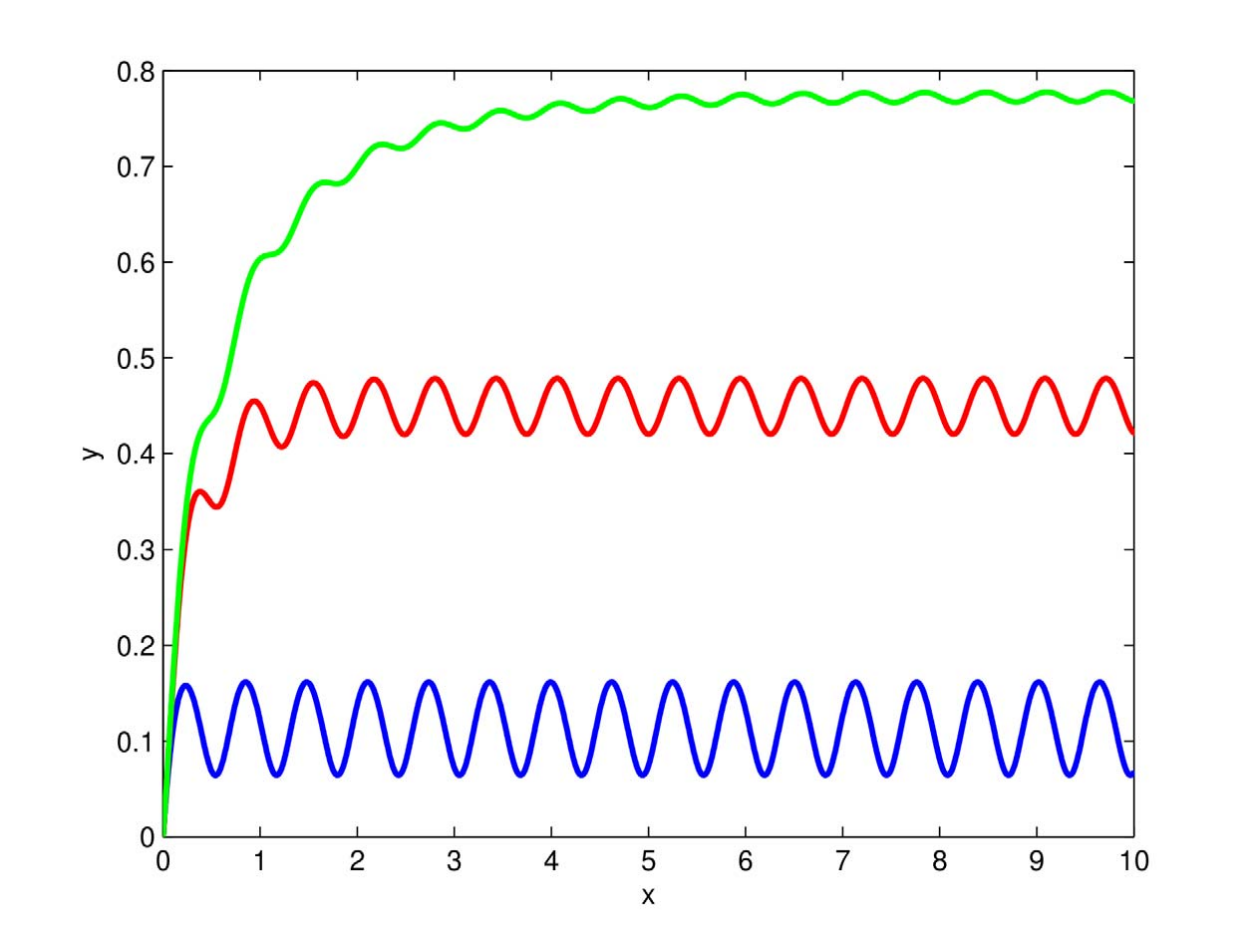
Entrainment of (9) to 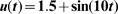. Time (minutes) on the 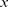-axis. The Figure shows the behavior of (9) for 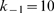 (blue), 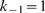 (green), 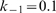 (red). Notice that an increase of 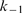, causes an increase of the contraction rate, hence trajectories converge faster to the system unique periodic attractor. The other system parameters are set to: 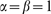, 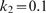

Figure 1 shows the behavior of the dynamical system (9), using two different values of 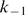. Notice that the asymptotic behavior of the system depends on the particular choice of the biochemical parameters being used. Furthermore, it is worth noticing here that the higher the value of 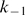, the faster will be the convergence to the attractor.

## Results

### Mathematical model and problem statement

We study a general externally-driven transcriptional module. We assume that the rate of production of a transcription factor 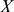 is proportional to the value of a time dependent input function 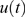, and 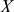 is subject to degradation and/or dilution at a linear rate. (Later, we generalize the model to also allow nonlinear degradation as well.) The signal 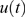 might be an external input, or it might represent the concentration of an enzyme or of a second messenger that activates 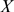. In turn, 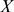 drives a downstream transcriptional module by binding to a promoter (or substrate), denoted by 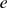 with concentration 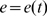. The binding reaction of 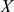 with 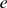 is reversible and given by:
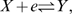
where 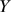 is the complex protein-promoter, and the binding and dissociation rates are 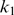 and 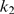 respectively. As the promoter is not subject to decay, its total concentration, 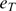, is conserved, so that the following conservation relation holds:
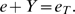
(11)We wish to study the behavior of solutions of the system that couples 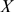 and 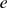, and specifically to show that, when the input 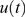 is periodic with period 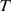, this coupled system has the property that all solutions converge to some globally attracting limit cycle whose period is also 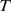.

**Figure 2:**
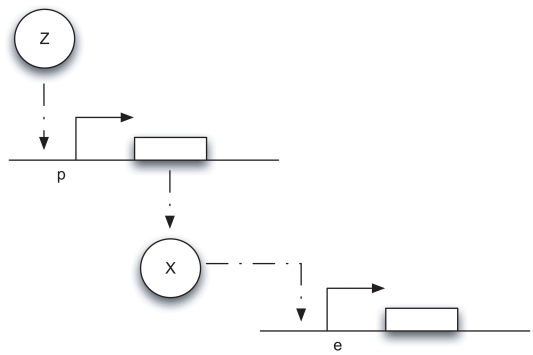
A schematic diagram of the transcriptional system modeled in (12). As explained in [29], the transcriptional component takes as input the concentration of protein 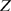 and gives as output the concentration of protein 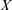. The downstream transcriptional module takes as input the concentration of protein 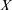.

Such transcriptional modules are ubiquitous in biology, natural as well as synthetic, and their behavior was recently studied in [29] in the context of “retroactivity” (impedance or load) effects. If we think of 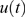 as the concentration of a protein 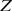 that is a transcription factor for 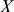, and we ignore fast mRNA dynamics, such a system can be schematically represented as in Figure 2, which is adapted from [29]. Notice that 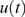 here does not need to be the concentration of a transcriptional activator of 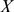 for our results to hold. The results will be valid for any mathematical model for the concentrations, 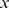, of 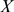 and 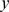, of 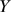 (the concentration of 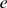 is conserved) of the form:
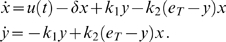
(12)


**Figure 3:**
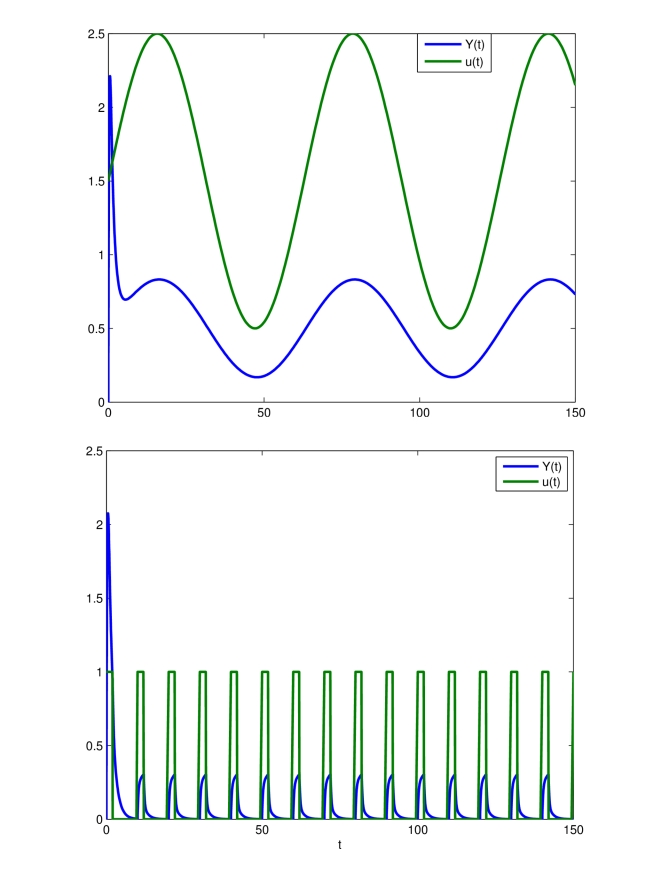
Entrainment of the transcriptional module (12). Time in minutes on the 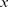-axis. The state of the system (green), 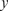, is entrained to both 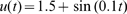 and to a repeating 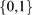 sequence. System parameters are set to: 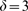, 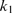 = 1, 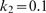.

An objective in this paper is, thus, to show that, when 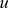 is a periodic input, all solutions of system (12) converge to a (unique) limit cycle (Figure 3). The key tool in this analysis is to show that uniform contractivity holds. Since in this example the input appears additively, uniform contractivity is simply the requirement that the unforced system (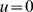) is contractive. Thus, the main step will be to establish the following technical result, see the Material and Methods:

**Proposition 1.** *The system*

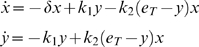

*where*

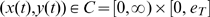
(13)
*for all* 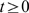 *, and* 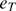 *,* 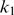 *,* 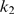 *, and* 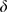 *are arbitrary positive constants, is contracting.*

Appealing to Theorem 2, we then have the following immediate Corollary:

**Proposition 2.** *For any given nonnegative periodic input* 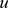 *of period* 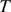 *, all solutions of system (12) converge exponentially to a periodic solution of period* 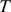.

In the following sections, we introduce a matrix measure that will help establish contractivity, and we prove Proposition 1. We will also discuss several extensions of this result, allowing the consideration of multiple driven subsystems as well as more general nonlinear systems with a similar structure. (A graphical algorithm to prove contraction of generic networks of nonlinear systems can also be found in [18] where this transcriptional module is also studied.)

### Proof of Proposition 1

We will use Theorem 2. The Jacobian matrix to be studied is:
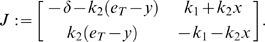
(14)As matrix measure, we will use the measure 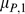 induced by the vector norm 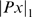, where 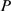 is a suitable nonsingular matrix. More specifically, we will pick 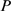 diagonal:
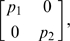
(15)where 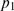 and 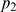 are two positive numbers to be appropriately chosen depending on the parameters defining the system.

It follows from general facts about matrix measures that
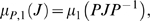
(16)where 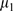 is the measure associated to the 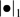 norm and is explicitly given by the following formula:
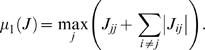
(17)Observe that, if the entries of 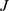 are negative, then asking that 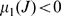 amounts to a column diagonal dominance condition. (The above formula is for real matrices. If complex matrices would be considered, then the term 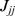 should be replaced by its real part 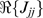.)

Thus, the first step in computing 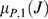 is to calculate 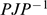:
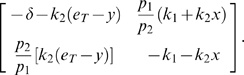
(18)Using (17), we obtain:
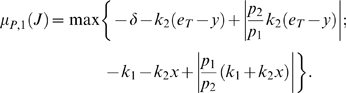
(19)Note that we are not interested in calculating the exact value for the above measure, but just in ensuring that it is negative. To guarantee that 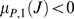, the following two conditions must hold:
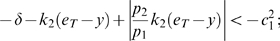
(20)

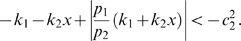
(21)Thus, the problem becomes that of checking if there exists an appropriate range of values for 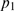, 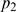 that satisfy (20) and (21) simultaneously.

The left hand side of (21) can be written as:
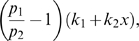
(22)which is negative if and only if 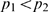. In particular, in this case we have:
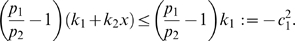
The idea is now to ensure negativity of (20) by using appropriate values for 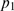 and 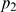 which fulfill the above constraint. Recall that the term 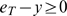 because of the choice of the state space (this quantity represents a concentration). Thus, the left hand side of (20) becomes
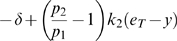
(23)The next step is to choose appropriately 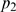 and 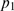 (without violating the constraint 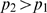). Imposing 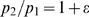, 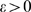, (23) becomes
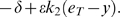
(24)Then, we have to choose an appropriate value for 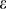 in order to make the above quantity uniformly negative. In particular, (24) is uniformly negative if and only if
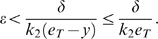
(25)We can now choose
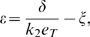
with 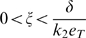. In this case, (24) becomes
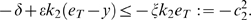
Thus, choosing 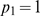 and 
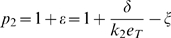
, with 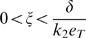, we have 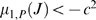. Furthermore, the contraction rate 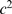, is given by:
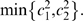
Notice that 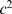 depends on both system parameters and on the elements 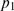, 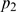, i.e. it depends on the particular metric chosen to prove contraction. This completes the proof of the Proposition.

### Generalizations

In this Section, we discuss various generalizations that use the same proof technique.

#### Assuming 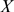 activation by enzyme kinetics

The previous model assumed that 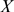 was created in proportion to the amount of external signal 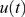. While this may be a natural assumption if 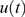 is a transcription factor that controls the expression of 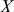, a different model applies if, instead, the “active” form 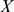 is obtained from an “inactive” form 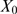, for example through a phosphorylation reaction which is catalyzed by a kinase whose abundance is represented by 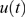. Suppose that 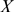 can also be constitutively deactivated. Thus, the complete system of reactions consists of
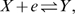
together with
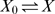
where the forward reaction depends on 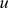. Since the concentrations of 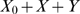 must remain constant, let us say at a value 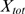, we eliminate 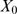 and have:
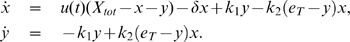
(26)


We will prove that if 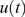 is periodic and positive, i.e. 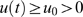, then a globally attracting limit cycle exists. Namely, it will be shown, after having performed a linear coordinate transformation, that there exists a negative matrix measure for the system of interest.

Consider, indeed, the following change of the state variables:
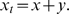
(27)The system dynamics then becomes:
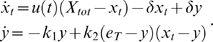
(28)As matrix measure, we will now use the measure 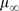 induced by the vector norm 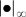. (Notice that this time, the matrix 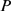 is the identity matrix).

Given a real matrix 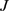, the matrix measure 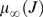 is explicitly given by the following formula (see e.g. [23]):
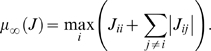
(29)(Observe that this is a row-dominance condition, in contrast to the dual column-dominance condition used for 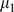.)

Differentiation of (28) yields the Jacobian matrix:
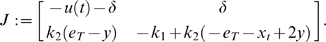
Thus, it immediately follow from (29) that 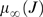 is negative if and only if:
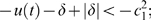
(30)

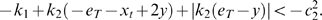
(31)The first inequality is clearly satisfied since by hypotheses both system parameters and the periodic input 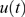 are positive. In particular, we have:
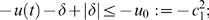
By using (27) (recall that 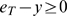), the right hand side of the second inequality can be written as:
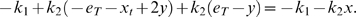
Since all system parameters are positive and 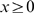, the above quantity is negative and upper bounded by 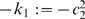.

Thus, we have that 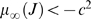, where:
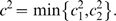
The contraction property for the system is then proved. By means of Theorem 2, we can then conclude that the system can be entrained by any periodic input.

**Figure 4:**
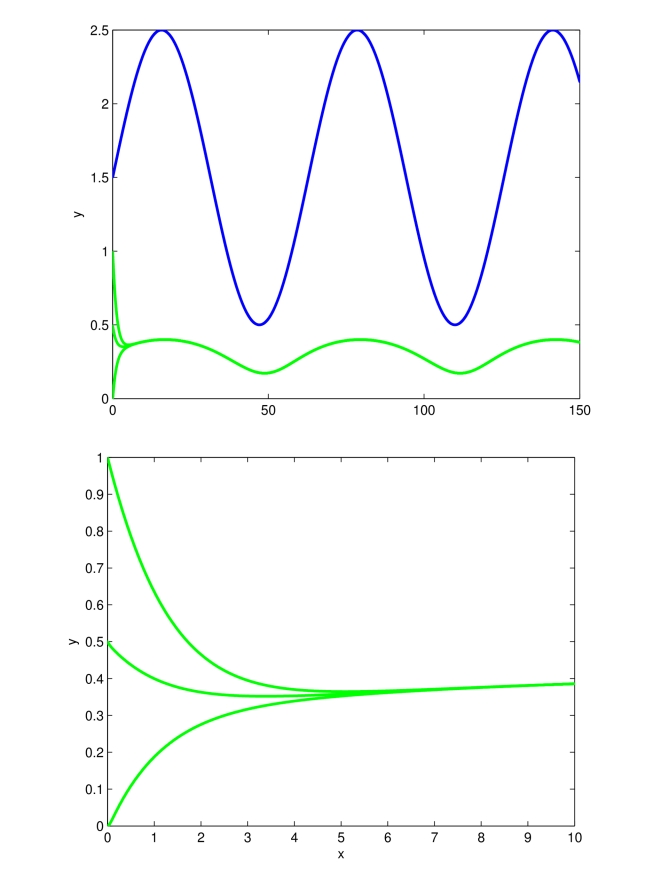
Entrainment of the transcriptional module (26). Time in minutes on the 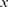-axis. The system state (green), 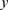, is entrained to the periodic input (blue): 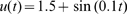. The zoom on 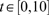 min highlights that trajectories starting from different initial conditions converge towards the attracting limit cycle. System parameters are set to: 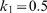, 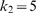, 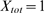, 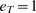, 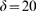.

Simulation results are presented in Figure 4, where the presence of a stable limit cycle having the same period as 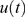 is shown.

#### Multiple driven systems

**Figure 5:**
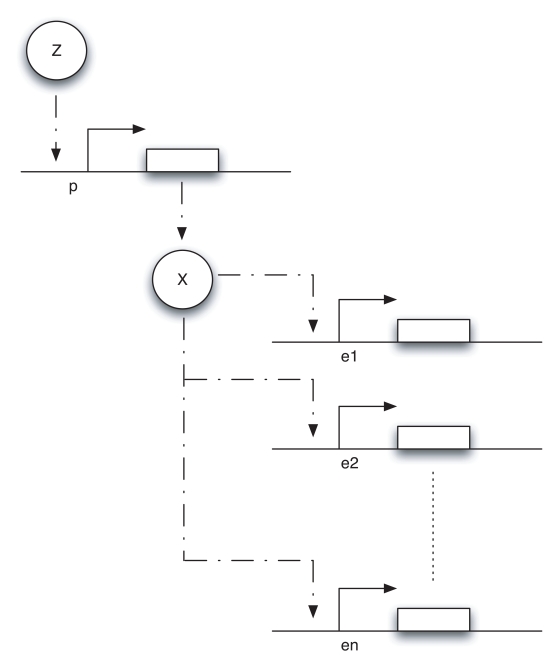
Multiple driven transcriptional modules. A schematic diagram of the transcriptional modules given in (12).

We may also treat the case in which the species 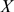 regulates multiple downstream transcriptional modules which act independently from each other, as shown in Figure 5. The biochemical parameters defining the different downstream modules may be different from each other, representing a situation in which the transcription factor 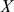 regulates different species. After proving a general result on oscillations, and assuming that parameters satisfy the retroactivity estimates discussed in [29], one may in this fashion design a single input-multi output module in which e.g. the outputs are periodic functions with different mean values, settling times, and so forth.

We denote by 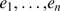 the various promoters, and use 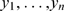 to denote the concentrations of the respective promoters complexed with 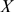. The resulting mathematical model becomes:
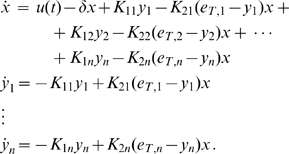
(32)


We consider the corresponding system with no input first, assuming that the states satisfy 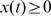 and 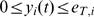 for all 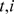.

Our generalization can be stated as follows:

**Proposition 3.** *System (32) with no input (i.e.* 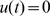 *) is contracting. Hence, if* 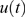 *is a non-zero periodic input, its solutions exponentially converge towards a periodic orbit of the same period as* 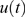.

*Proof.* We only outline the proof, since it is similar to the proof of Proposition 2. We employ the following matrix measure:
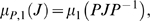
(33)where
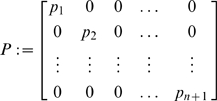
(34)and the scalars 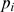 have to be chosen appropriately (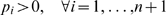).

In this case,
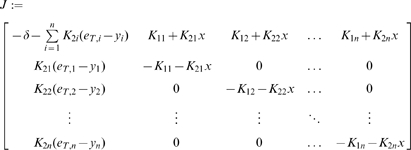
(35)and
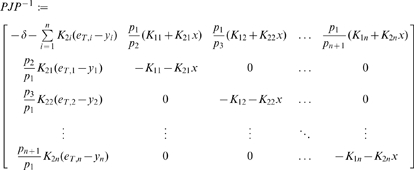
(36)


Hence, the 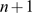 inequalities to be satisfied are:
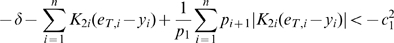
(37)and
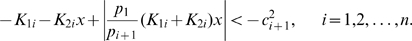
(38)


Clearly, the set of inequalities above admits a solution. Indeed, the left hand side of (38) can be recast as
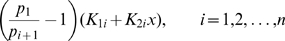
which is negative definite if and only if 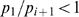 for all 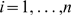. Specifically, in this case we have
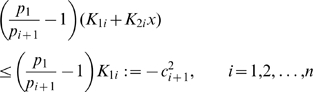
Also, from (37), as 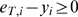 for all 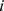, we have that (37) can be rewritten as:
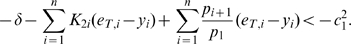
Since 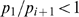, we can impose 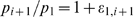 (with 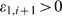) and the above inequality becomes
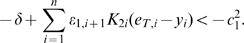
Clearly, such inequality is satisfied if we choose 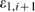 sufficiently small; namely:
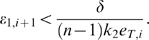
Following a similar derivation to that of the previous Section, we can choose
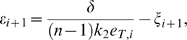
with 
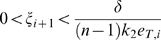
. In this case, we have:
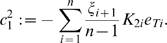
Thus, 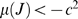, where
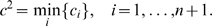
The second part of the Proposition is then proved by applying Theorem 2.

**Figure 6:**
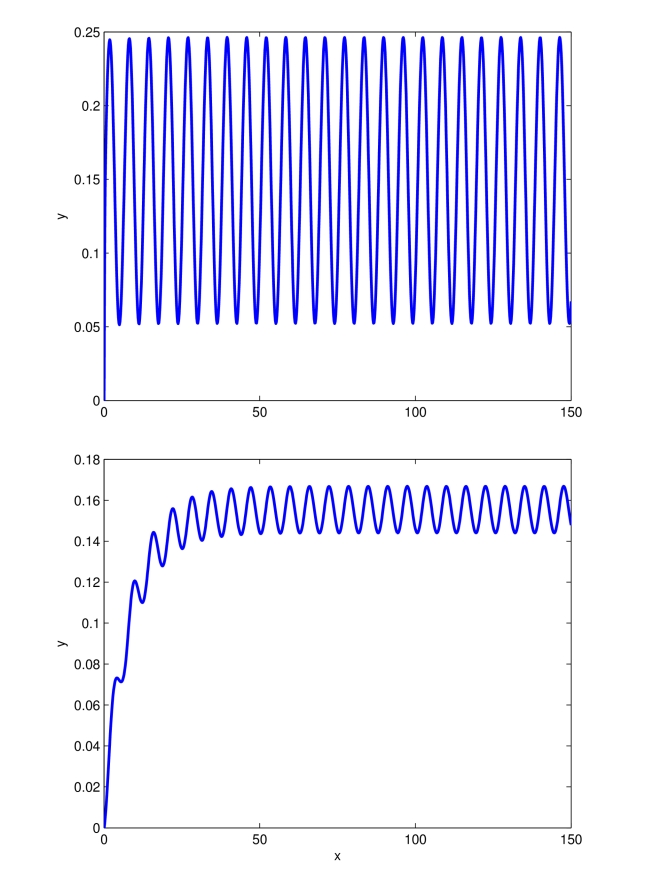
Entrainment of two-driven transcriptional modules. Time in minutes on the 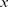-axis. Outputs 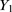 (top) and 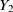 (bottom) of two transcriptional modules driven by the external periodic input 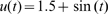. The parameters are set to: 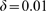, 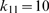, 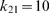, 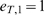 for module 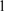 and 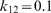, 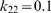, 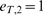 for module 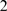.

In Figure 6 the behavior of two-driven downstream transcriptional modules is shown. Notice that both the downstream modules are entrained by the periodic input 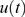, but their steady state behavior is different.

Notice that, by the same arguments used above, it can be proven that
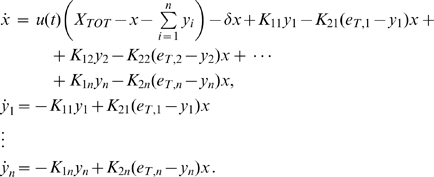
(39)is contracting.

#### Transcriptional cascades

**Figure 7:**
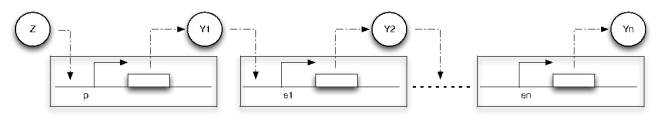
Transcriptional cascade discussed in the text. Each box contains the transcriptional module described by (12).

A cascade of (infinitesimally) contracting systems is also (infinitesimally) contracting [6], [30] (see Materials and Methods for an alternative proof). This implies that any transcriptional cascade, will also give rise to a contracting system, and, in particular, will entrain to periodic inputs. By a transcriptional cascade we mean a system as shown in Figure 7. In this figure, we interpret the intermediate variables 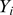 as transcription factors, making the simplifying assumption that TF concentration is proportional to active promoter for the corresponding gene. (More complex models, incorporating transcription, translation, and post-translational modifications could themselves, in turn, be modeled as cascades of contracting systems.)

#### More abstract systems

We can extend our results even further, to a larger class of nonlinear systems, as long as the same general structure is present. This can be useful for example to design new synthetic transcription modules or to analyze the entrainment properties of general biological systems. We start with a discussion of a two dimensional system of the form:
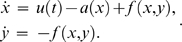
(40)In molecular biology, 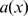 would typically represent a nonlinear degradation, for instance in Michaelis-Menten form, while the function 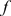 represents the interaction between 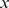 and 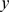. The aim of this Section is to find conditions on the degradation and interaction terms that allow one to show contractivity of the unforced (no input 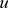) system, and hence existence of globally attracting limit cycles.

We assume that the state space 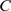 is compact (closed and bounded) as well as convex. Since the input appears additively, we must prove contractivity of the unforced system.

**Theorem 3.** *System (40), without inputs* 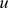 *, evolving on a convex compact subset of phase space is contracting, provided that the following conditions are all satisfied, for each* 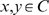 *:*

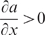
;

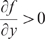
;

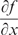
 *does not change sign;*

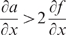
.

Notice that the last condition is automatically satisfied if 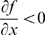, because 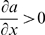.

As before, we prove contraction by constructing an appropriate negative measure for the Jacobian of the vector field. In this case, the Jacobian matrix is:
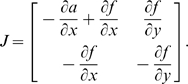
(41)Once again, as matrix measure we will use:
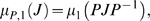
(42)with 
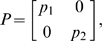
(43)and 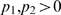 appropriately chosen.

Using (42) we have
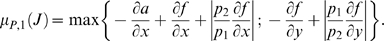
(44)Following the same steps as the proof of Proposition 1, we have to show that:
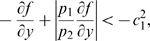
(45)

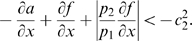
(46)


Clearly, if 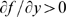 for every 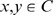 and 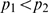, the first inequality is satisfied, with
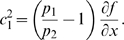
To prove the theorem we need to show that there exists 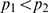 and 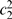 satisfying (46). For such inequality, since 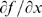 does not change sign in 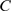 by hypothesis, we have two possibilities:

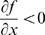
, 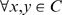;

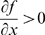
, 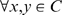.

In the first case, the right hand side of (46) becomes
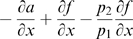
(47)Choosing 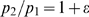, with 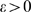, we have:
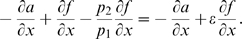
Specifically, if we now pick
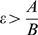
where 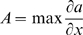 and 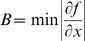, we have that the above quantity is uniformly negative definite, i.e.
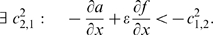
In the second case, the right hand side of (46) becomes
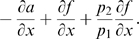
(48)Again, by choosing 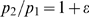, with 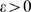, we have the following upper bound for the expression in (48):
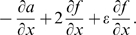
(49)Thus, it follows that 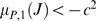 provided that the above quantity is uniformly negative definite. Since, by hypotheses,
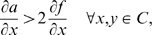
(50)then 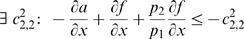. The proof of the Theorem is now complete.

From a biological viewpoint, the hardest hypothesis to satisfy in Theorem 3 might be that on the derivatives of 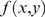. However, it is possible to relax the hypothesis on 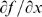 if the rate of change of 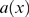 with respect to 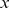, i.e. 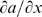, is sufficiently larger than 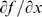. In particular, the following result can be proved.

**Theorem 4.** *System (40), without inputs* 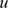 *, evolving on a convex compact set, is contractive provided that:*

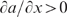
, 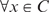;

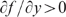
, 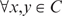;

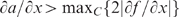
.

*Proof.* The proof is similar to that of Theorem 3. In particular, we can repeat the same derivation to obtain again inequality (46). Thence, as no hypothesis is made on the sign of 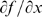, choosing 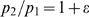 we have
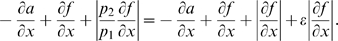
(51)Thus, it follows that, if 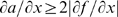, then 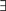 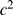 such that 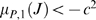, implying contractivity. The above condition is satisfied by hypotheses, hence the theorem is proved.

#### Remarks

Theorems 3 and 4 show the possibility of designing with high flexibility the self-degradation and interaction functions for an input-output module.

This flexibility can be further increased, for example in the following ways:

- Results similar to that of the above Theorems can be derived (and also extended) if some self degradation rate for 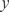 is present in (40), i.e.
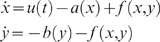
(52)with 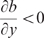.
- Theorem 3 and Theorem 4 can also be extended to the case in which the 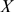-module drives more than one downstream transcriptional modules.

### Applications to synthetic biology

We introduced above a methodology for checking if a given transcriptional module can be entrained to some periodic input. The aim of this section is to show that our methodology can serve as an effective tool for designing synthetic biological circuits that are entrained to some desired external input.

In particular, we will consider the synthetic biological oscillator known as the Repressilator [31], for which an additional coupling module has been recently proposed in [32]. A numerical investigation of the synchronization of a network of non-identical Repressilators was independently reported in [33].

We will show that our results can be used to isolate a set of biochemical parameters for which one can guarantee the entrainment to any external periodic signal of this synthetic biological circuit. In what follows, we will use the equations presented in [32] to model the Repressilator and the additional coupling model.

#### Entrainment using an intra-cellular auto-inducer

**Figure 8:**
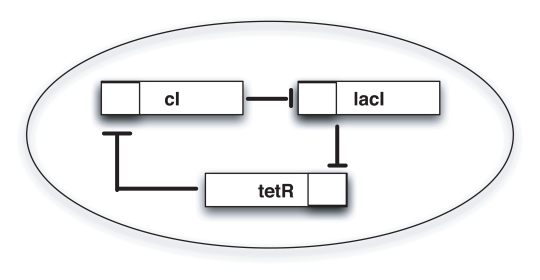
The Repressilator circuit. A schematic representation of the three-genes Repressilator circuit.

The Repressilator is a synthetic biological circuit that consists of three genes that inhibit each other in a cyclic way [31]. As shown in Figure 8, gene *lacI* (associated to the state variable 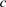 in the model) expresses protein LacI (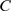), which inhibits the transcription of gene *tetR* (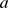). This translates into protein TetR (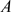), which inhibits transcription of gene *cI* (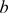). Finally, the protein CI (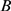), translated from *cI*, inhibits expression of *lacI*, completing the cycle.

**Figure 9:**
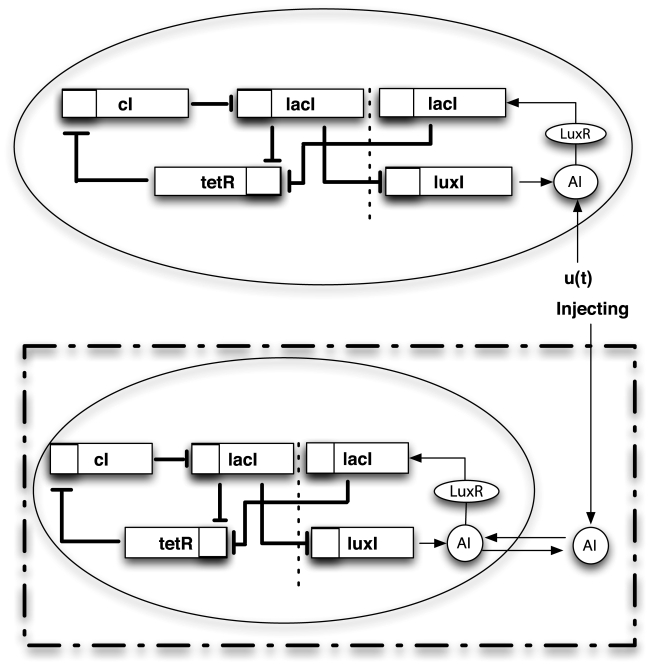
Modular addition to the Repressilator circuit. This module is used for forcing the original circuit with some external signal (represented by an extra-cellular molecule in the bottom panel).

In Figure 9 a modular addition to the three-genes circuit is presented. The module was first presented in [32] and makes the Repressilator circuit sensitive to the concentration of the auto-inducer (labeled as 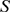 in the model) which is a small molecule that can pass through the cell membrane. Specifically, the module makes use of two proteins: (i) LuxI, which synthesizes the auto-inducer; (ii) LuxR, with which the auto-inducer synthesized by LuxI forms a complex that activates the transcription of various genes.

We model the above circuit with the simplified set of differential equations proposed in [32]. Specifically, the dynamics of the *mRNA*s are
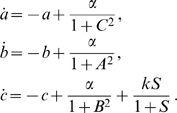
(53)


Notice that the above equations are dimensionless. This is done by: (i) measuring time in units of *mRNA* lifetime (which is assumed equal for the three genes), and (ii) expressing the protein levels in units of their Michaelis constant. The parameter 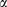 represents the dimensionless transcription rate in the absence of self-repression, while 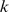 denotes the maximum contribution of the auto-inducer to the expression of *lacI*.

The dynamics of the proteins are described by
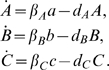
(54)The parameters 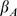, 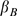, 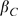 represent the ratios between the *mRNA*s and the respective proteins' lifetimes and 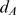, 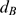, 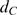 represent the protein decay rate.

The last differential equation of the model from [32] keeps track of the evolution of the intra-cellular auto-inducer. It is assumed that the proteins TetR and LuxI have equal lifetimes. This in turn implies that the dynamics of such proteins are identical, and hence one uses the same variable to describe both protein concentrations. Thus, the dynamics of the auto-inducer are given by:
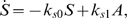
where 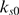 is the rate of degradation of 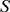.

We now model the forcing on the intracellular auto-inducer concentration by adding an external input 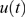 to the above dynamical equation. The equation for 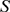 becomes:
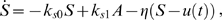
(55)where 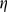 can be thought as a diffusion rate.

We will now use the analytical methodology developed in the previous sections, to properly tune the biochemical parameters of the Repressilator circuit, whose mathematical model consists of the set of differential equations (53), (54), (55), so that it shows entrainment to the periodic input 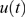. That is, the measured output (e.g. 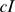), oscillates asymptotically with a period equal to that of 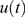. Of course, the periodic orbit of the output will depend on the particular choice of the parameters.

In what follows, we assume that all the system parameters can be varied except for the self-degradations that we assume to be fixed as, in practice, they are difficult to modify.

In this case, the Jacobian matrix to be studied is
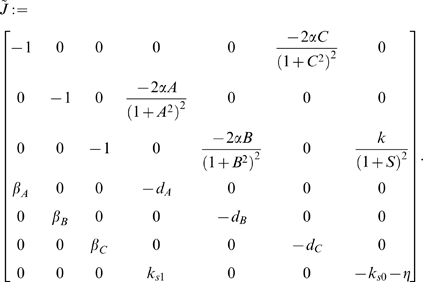
(56)


The matrix measure that we will use to prove contraction is
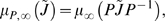
where 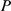 is a 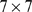 diagonal matrix having on the main diagonal the positive arbitrary scalars 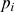. Computation of 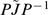 yields
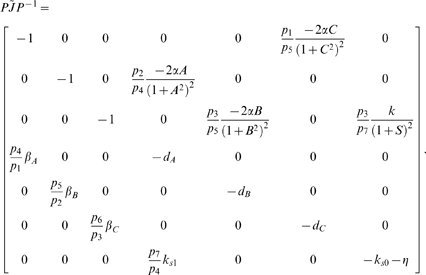
(57)Thus, from the definition of 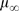 given in (29), we have that there exists some 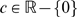 such that 
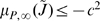
, 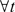 if and only if there exists a set of scalars 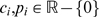, 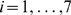, such that
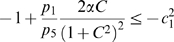
(58a)

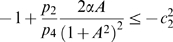
(58b)

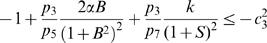
(58c)

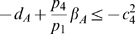
(58d)

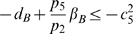
(58e)

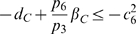
(58f)

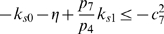
(58g)


It is easy to check that the nonlinear terms in the above equations satisfy the following inequalities:
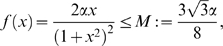
and
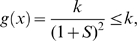
for all 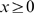. Hence, the system of inequalities (58a)–(58g) are satisfied, if the following set is fulfilled:
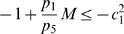
(59a)

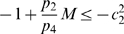
(59b)

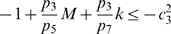
(59c)

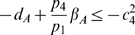
(59d)

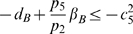
(59e)

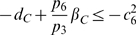
(59f)

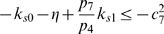
(59g)


**Figure 10:**
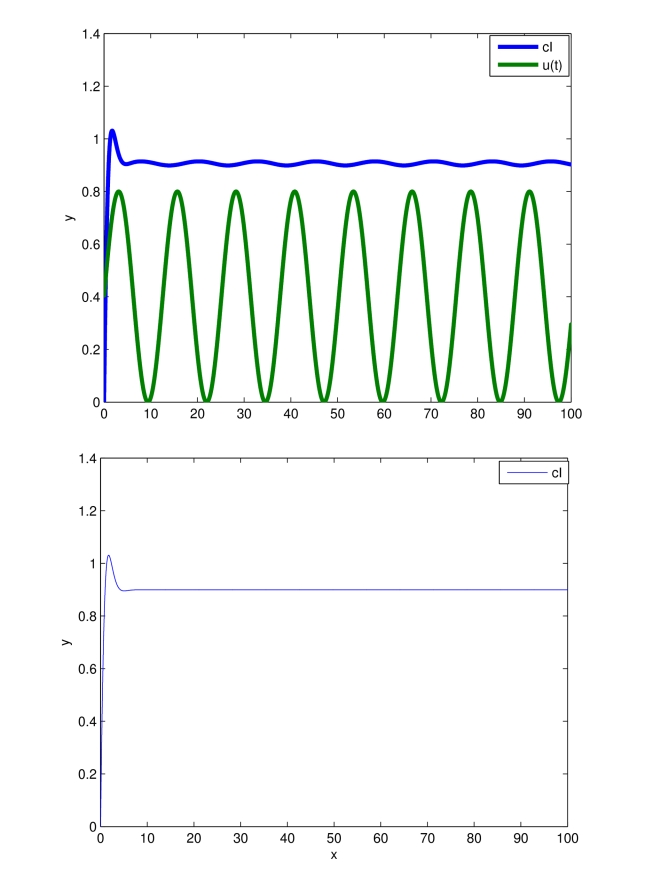
Simulation of the Repressilator model described by (53), (54), (55). Time (minutes) on the 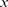-axis. Behavior of 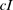 when the input 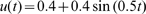 is applied. Notice that when no forcing is present 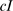 converges to a non oscillatory regime behavior. System parameters are tuned in order to satisfy (72). Specifically: 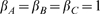, 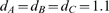, 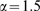, 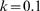, 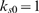, 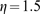, 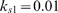.

The system can then be proved to be contracting for a given set of biochemical parameters, if there exists a set of scalars 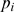, 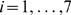 satisfying the above inequalities. For example, if the repressilator parameters are chosen so that
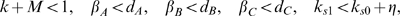
(60)then it is trivial to prove that, for any constant value 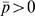, the set of scalars 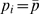, for 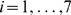, satisfies (59a)–(59g). Indeed, in Figure 10 we provide a set of biochemical parameters for which the circuit is contracting and shows entrainment to the periodic input 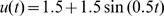. (These parameters, except for the maximal transcription rate 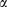, are in the same ranges as those used in [31], [32]. These parameters are also close to those used in [33] and [34]. The reason for picking an 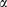 much smaller than in [32], is that we need to slow down transcription so as to eliminate intrinsic oscillations; otherwise the entrainment effect cannot be shown. This lowering of 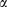 by two orders of magnitude is also found in other works, for example in [35], where the same model is studied, with 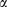 somewhat larger but of the same order of magnitude as here.)

Note that using the set of inequalities (59a)–(59g) as a guideline, it is possible to find other parameter regions where the system is still contracting but exhibit some other desired properties. For instance, to tune (e.g. increase) the amplitude of the output oscillations shown in Figure 10, a possible approach can be that of increasing the biochemical parameter 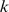 so as to make stronger the effect of the auto-inducer on the dynamics of the gene 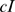 (variable 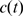 in the model).

Again we can prove that the set of inequalities (59a)–(59g) is satisfied for 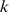 arbitrarily large, if we set 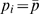, for 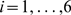 and choose 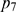 such that
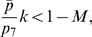
and
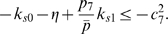
Now, due to biochemical constraints the parameter 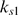 is considerably smaller than 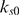 and 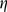 (in our simulations the ratio is of about two orders of magnitude). Therefore, whatever the value of 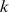, it suffices to set 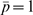 and 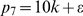, with 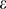 being a positive arbitrary constant, to get a solution to (59a)–(59g) and hence guarantee the system to be contracting.

**Figure 11:**
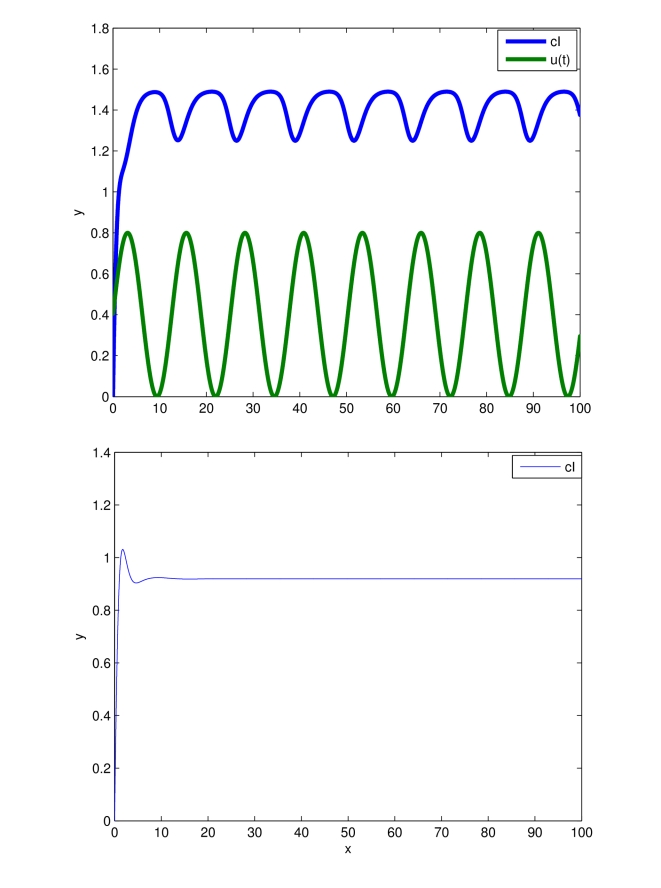
Increasing the amplitude of oscillations for the model described by (53), (54), (55). Time (minutes) on the 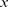-axis. Behavior of 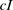 when: (i) the input 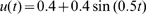 is applied; (ii) no forcing is present. System parameters are the same as that used in Figure 10, except 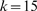.

Figure 11 shows the behavior of the system output with the modified parameters confirming that with this choice of parameters the oscillation amplitude is indeed larger as expected.

Observe the *nonlinear character of the oscillation* depicted in Figure 11, which is reflected in the lack of symmetry in the behavior at minima and maxima of *cI* 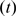. Our theory predicts the existence (and uniqueness) of such a nonlinear oscillations. None of the usual techniques, based on linear analysis, can explain such behavior.

#### Entrainment using an extra-cellular auto-inducer

We now consider the case in which the extracellular auto-inducer can change due to an external signal as well as diffusion from intracellular auto-inducer, as represented in Figure 9. A new variable must be introduced, to keep track of the extracellular auto-inducer concentration. The only difference in the new model with respect to the previous one is that the differential equation for 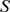 becomes:
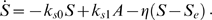
(61)Notice that the parameter 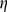 measures the diffusion rate of the auto-inducer across the cell membrane, i.e. 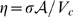, with 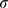 representing the membrane permeability, 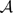 its surface area and 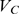 the cell volume. In the above equation, 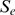 denotes the concentration of the extra-cellular auto-inducer, whose dynamics are given by:
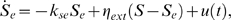
(62)where 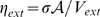, with 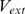 denoting the total extracellular volume, while 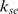 stands for the decay rate.

In analogy with the previous section, we will ensure entrainment of the dynamical system consisting of (53), (54), (61), (62), by tuning the biochemical parameters of this new circuit. Again, the guidelines for engineering the parameters will be provided by the tools developed in the previous sections.

Following the schematic of the previous section, we will prove that there exists 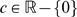 and a 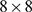 constant diagonal matrix 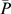, such that 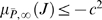, where 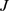 is the system Jacobian.

If we denote with 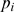, 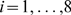 the diagonal elements of 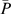, we obtain the following block-structure for the matrix 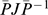:
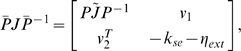
(63)where 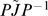 is given in (57) and:
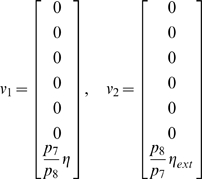
(64)


Thus, we have that 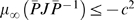 if and only if there exist some 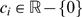, 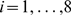 such that inequalities (58a)–(58f) are all satisfied and additionally:
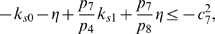
(65a)

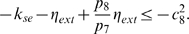
(65b)


Again, we can find sets of biochemical parameters in order to satisfy the above inequalities and hence ensure global entrainment of the circuit to some external input. For example, if we set
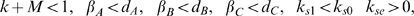
(66)then, as in the previous section, it is trivial to show that setting all 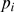 to the same identical value satisfies the set of inequality required to prove contraction and hence guarantees entrainment. Notice that the last constraint in (66) is automatically satisfied by the physical (i.e. positivity) constraints on the system parameters.

**Figure 12:**
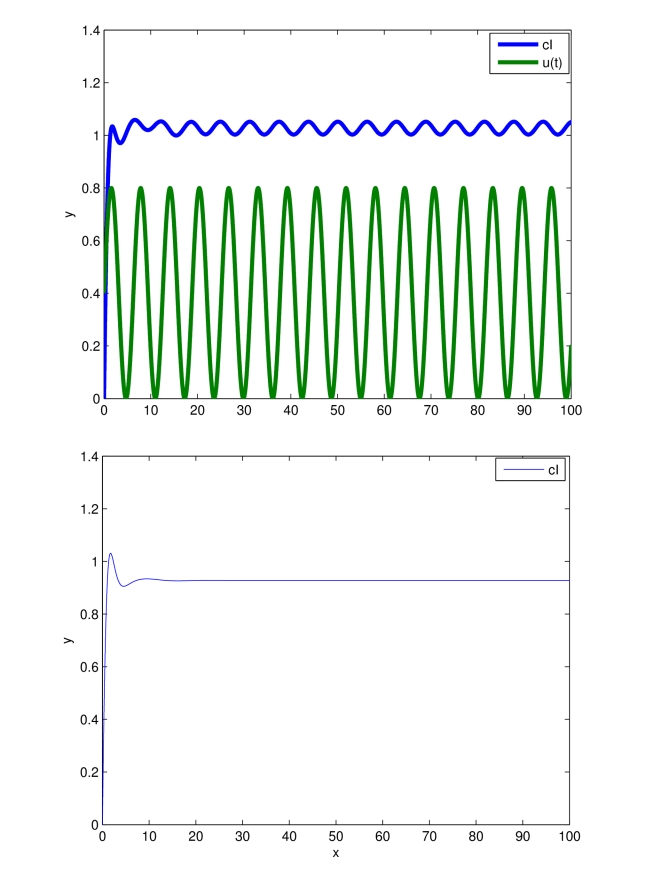
Simulation of the Repressilator forced by some extra-cellular molecule. Time (minutes) on the 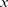-axis. Behavior of 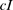 when the input 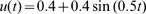 is applied. Notice that when no forcing is present, the steady state behavior is non-oscillatory. System parameters are: 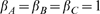, 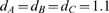, 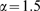, 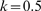, 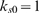, 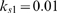.

In Figure 12, the behavior of the circuit is shown with the parameters chosen so as to satisfy the constraints given in (66).

#### Entraining a population of Repressilators

Consider, now, a population of 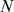 Repressilator circuits, which are coupled by means of an auto-inducer molecule. We can think of such a network as having an all-to-all topology, with the coupling given by the concentration of the extracellular auto-inducer, 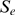. The aim of this section is to show that the methodology proposed here can also be used as an effective tool to guarantee the synchronization of an entire population of biochemical oscillators onto some entraining external periodic input.

We denote with the subscript 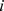 the state variables of the 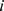-th circuit in the network, which is modelled using the equations reported in [32] as:
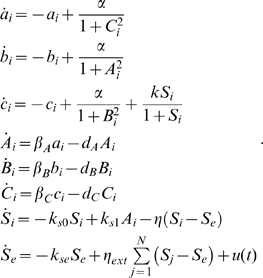
(67)


**Figure 13:**
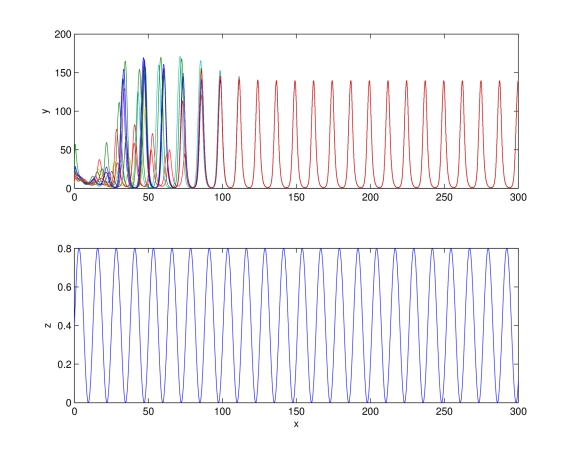
Synchronization of Repressilators. Behavior of a population of Repressilator modeled as in (80). Time (minutes) on 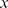-axs. Notice that all the circuits synchronize with a steady-state evolution having the same period as 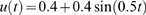. System parameters are chosen as in Figure 11, with 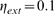.

Figure 13 shows a simulation of a population of Repressilators modeled as in (67), with biochemical parameters tuned as in the previous Section: all the circuits composing the network evolve asymptotically towards the same synchronous evolution, which has period equal to that of the input signal 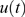. The interested reader is referred to the Materials and Methods for the proof.

## Materials and Methods

All simulations are performed in MATLAB (Simulink), Version 7.4, with variable step ODE solver ODE23t. Simulink models are available upon request. The proofs of the results are as follows.

### 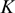-reachable sets

We will make use of the following definition:

**Definition 1.** *Let* 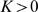 *be any positive real number. A subset* 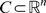 *is* 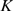-reachable *if, for any two points* 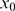 *and* 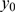 *in* 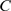 *there is some continuously differentiable curve* 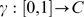 *such that:*

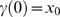
,

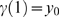
 *and*

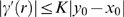
, 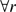.

For convex sets 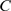, we may pick 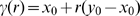, so 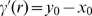 and we can take 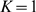. Thus, convex sets are 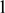-reachable, and it is easy to show that the converse holds as well.

Notice that a set 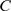 is 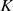-reachable for some 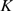 if and only if the length of the geodesic (smooth) path (parametrized by arc length), connecting any two points 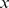 and 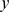 in 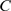, is bounded by some multiple 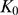 of the Euclidean norm, 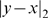. Indeed, re-parametrizing to a path 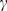 defined on 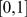, we have:
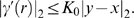
Since in finite dimensional spaces all the norms are equivalent, then it is possible to obtain a suitable 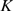 for Definition 1.

**Remark 1.** *The notion of* 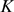 *-reachable set is weaker than that of convex set. Nonetheless, in Theorem 5, we will prove that trajectories of a smooth system, evolving on a* 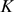 *-reachable set, converge towards each other, even if* 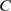 *is not convex. This additional generality allows one to establish contracting behavior for systems evolving on phase spaces exhibiting “obstacles”, as are frequently encountered in path-planning problems, for example. A mathematical example of a set with obstacles follows.*

**Example 1.** *Consider the two dimensional set,* 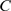 *, defined by the following constraints:*

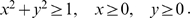

*Clearly,* 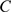 *is a non-convex subset of* 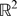 *. We claim that* 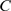 *is* 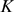 *-reachable, for any positive real number* 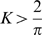 *. Indeed, given any two points* 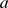 *and* 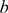 *in* 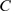 *, there are two possibilities: either the segment connecting* 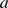 *and* 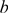 *is in* 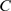 *, or it intersects the unit circle. In the first case, we can simply pick the segment as a curve (* 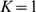 *). In the second case, one can consider a straight segment that is modified by taking the shortest perimeter route around the circle; the length of the perimeter path is at most* 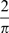 *times the length of the omitted segment. (In order to obtain a differentiable, instead of merely a piecewise-differentiable, path, an arbitrarily small increase in* 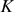 *is needed.)*

### Proof of Theorem 1

We now prove the main result on contracting systems, i.e. Theorem 1, under the hypotheses that the set 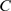, i.e. the set on which the system evolves, is 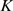-reachable.

**Theorem 5.** *Suppose that* 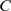 *is a* 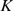 *-reachable subset of* 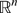 *and that* 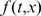 *is infinitesimally contracting with contraction rate* 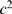 *. Then, for every two solutions* 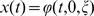 *and* 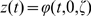 *it holds that:*

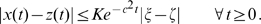
(68)


*Proof.* Given any two points 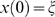 and 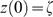 in 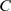, pick a smooth curve 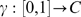, such that 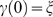 and 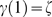. Let 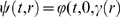, that is, the solution of system (1) rooted in 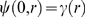, 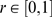. Since 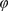 and 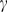 are continuously differentiable, also 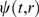 is continuously differentiable in both arguments. We define
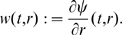
It follows that
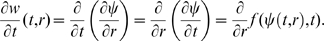
Now,
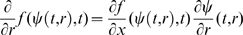
so, we have:
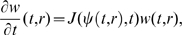
(69)where 
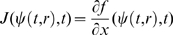
. Using Coppel's inequality [28], yields
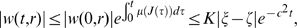
(70)

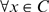
, 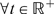, and 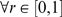. Notice the Fundamental Theorem of Calculus, we can write
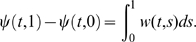
Hence, we obtain
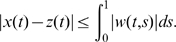
Now, using (70), the above inequality becomes:
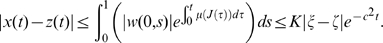
The Theorem is then proved.

**Proof of Theorem 1.** The proof follows trivially from Theorem 5, after having noticed that in the convex case, we may assume 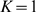.

### Proof of Theorem 2

In this Section we assume that the vector field 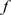 is 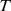-periodic and prove Theorem 2.

Before starting with the proof of Theorem 2 we make the following:

**Remark 2.** *Periodicity implies that the initial time is only relevant modulo* 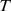 *. More precisely:*

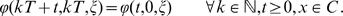
(71)
*Indeed, let* 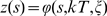 *,* 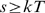 *, and consider the function* 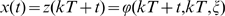 *, for* 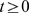 *. So, *

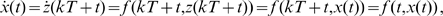

*where the last equality follows by* 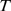 *-periodicity of* 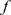 *. Since* 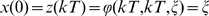 *, it follows by uniqueness of solutions that* 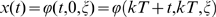 *, which is (71). As a corollary, we also have that*

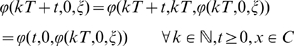
(72)
*where the first equality follows from the semigroup property of solutions (see e.g.* [*21*] *), and the second one from (71) applied to* 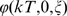 *instead of* 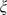.

Define now
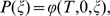
where 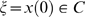. The following Lemma will be useful in what follows.

**Lemma 1.** 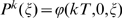 *for all* 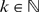 *and* 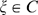.

*Proof.* We will prove the Lemma by recursion. In particular, the statement is true by definition when 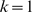. Inductively, assuming it true for 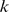, we have:
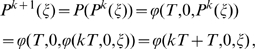
as wanted.

**Theorem 6.** *Suppose that:*

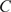
 *is a closed* 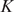 *-reachable subset of* 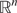 *;*

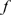
 *is infinitesimally contracting with contraction rate* 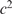 *;*

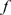
 *is* 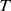 *-periodic;*

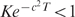
.

*Then, there is an unique periodic solution* 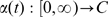 *of (1) having period* 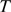 *. Furthermore, every solution* 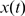 *, such that* 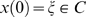 *, converges to* 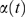 *, i.e.* 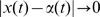 *as* 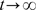.

*Proof.* Observe that 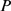 is a contraction with factor 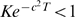: 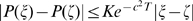 for all 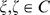, as a consequence of Theorem 5. The set 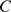 is a closed subset of 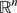 and hence complete as a metric space with respect to the distance induced by the norm being considered. Thus, by the contraction mapping theorem, there is a (unique) fixed point 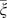 of 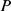. Let 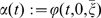. Since 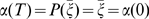, 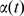 is a periodic orbit of period 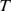. Moreover, again by Theorem 5, we have that 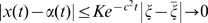. Uniqueness is clear, since two different periodic orbits would be disjoint compact subsets, and hence at positive distance from each other, contradicting convergence. This completes the proof.

**Proof of Theorem 2.** It will suffice to note that the assumption 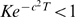 in Theorem 6 is automatically satisfied when the set 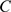 is convex (i.e. 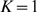) and the system is infinitesimally contracting.

Notice that, even in the non-convex case, the assumption 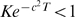 can be ignored, if we are willing to assert only the existence (and global convergence to) a unique periodic orbit, with some period 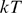 for some integer 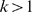. Indeed, the vector field is also 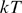-periodic for any integer 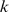. Picking 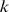 large enough so that 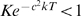, we have the conclusion that such an orbit exists, applying Theorem 6.

### Cascades

In order to show that cascades of contracting systems remain contracting, it is enough to show this, inductively, for a cascade of two systems.

Consider a system of the following form:
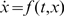


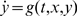
where 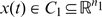 and 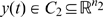 for all 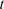 (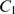 and 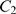 are two 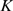-reachable sets). We write the Jacobian of 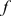 with respect to 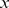 as 
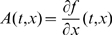
, the Jacobian of 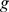 with respect to 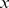 as 
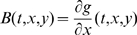
, and the Jacobian of 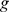 with respect to 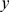 as 
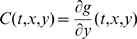
,

We assume the following:

1. The system 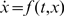 is infinitesimally contracting with respect to some norm (generally indicated as 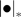), with some contraction rate 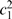, that is, 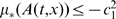 for all 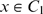 and all 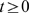, where 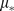 is the matrix measure associated to 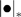.
2. The system 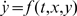 is infinitesimally contracting with respect to some norm (which is, in general different from 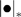, and is denoted by 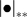), with contraction rate 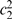, when 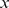 is viewed a a parameter in the second system, that is, 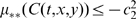 for all 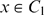, 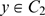 and all 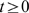, where 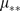 is the matrix measure associated to 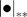.
3. The mixed Jacobian 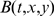 is bounded: 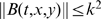, for all 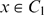, 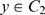 and all 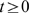, for some real number 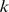, where “
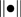
” is the operator norm induced by 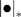 and 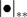 on linear operators 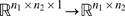. (All norms in Euclidean space being equivalent, this can be verified in any norm.)

We claim that, under these assumptions, the complete system is infinitesimally contracting. More precisely, pick any two positive numbers 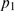 and 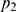 such that
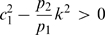
and let
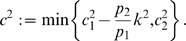
We will show that 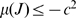, where 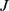 is the full Jacobian:
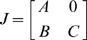
(73)with respect to the matrix measure 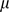 induced by the following norm in 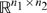:
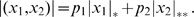
Since
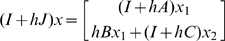
for all 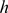 and 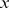, we have that, for all 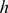 and 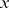:
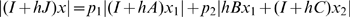


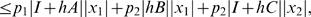
where from now on we drop subscripts for norms. Pick now any 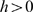 and a unit vector 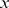 (which depends on 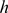) such that 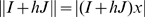. Such a vector 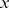 exists by the definition of induced matrix norm, and we note that 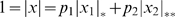, by the definition of the norm in the product space. Therefore:
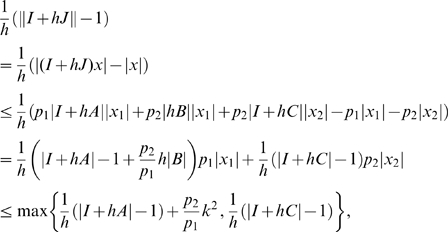
where the last inequality is a consequence of the fact that 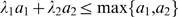 for any nonnegative numbers with 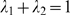 (convex combination of the 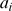
's). Now taking limits as 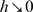, we conclude that
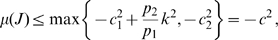
as desired.

### Entraining a population of Repressilators: proof

The general principle that we apply to prove entrainment of a population of Repressilators is as follows.

Assume that the cascade system
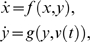
(74)with 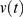 being an exogenous input, satisfies the contractivity assumptions of the above Section. Then, consider the interconnection of 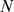 identical systems which interact through the variable 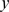 as follows:
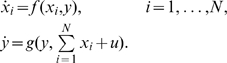
(75)Suppose that 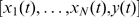 is a solution of (75) defined for all 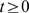, for some input 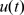. Then, we have the synchronization condition: 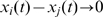, as 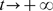.

Indeed, we only need to observe that every pair 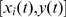 is a solution of (74) with the same input 
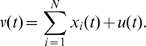
Furthermore, if 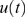 is a 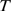-periodic function, the 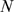 interconnected dynamical systems synchronize onto a 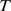-periodic trajectory.

The above principle can be immediately applied to prove that synchronization onto a 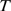-periodic orbit is attained for the Repressilator circuits composing network (67) (see also [19]).

Specifically, let 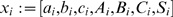 and 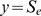; we have that 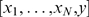 is a solution of (67). We notice that any pair 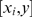 is a solution of the following cascade system
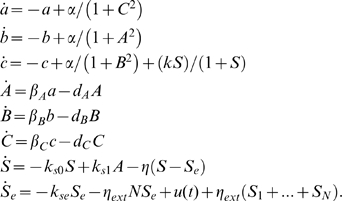
(76)Thus, as shown above, contraction of (76) implies synchronization of (67). Differentiation of (76) yields the Jacobian matrix
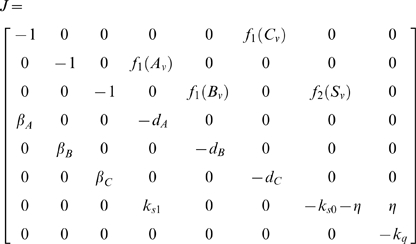
(77)where 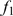 and 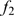 denote the partial derivatives of decreasing and increasing Hill functions with respect to the state variable of interest and 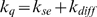, 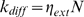.

Note that the Jacobian matrix 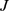 has the structure of a cascade, i.e.
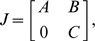
with:
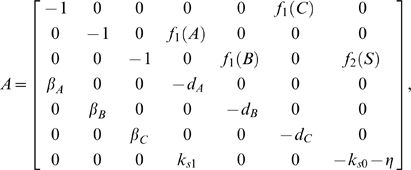


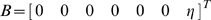
, 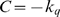. Thus, to prove contraction of the virtual system (76) it suffices to prove that there exist two matrix measures, 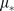 and 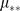 such that:

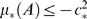
;

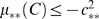
;

where 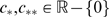. Clearly, since 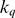 is a positive real parameter, the second condition above is satisfied (with 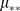 being any matrix measure). Now, notice that matrix 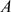 has the same form as the Jacobian matrix of the Repressilator circuit (56). Hence, if the parameters of the Repressilator are chosen so that they satisfy (66), then there exist a set of positive real parameters 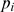, 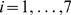, such that 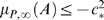 (that is, the first condition above is also satisfied with 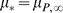).

Thus, we can conclude that (76) is contracting. Furthermore, all the trajectories of the virtual system converge towards a 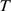-periodic solution (see Theorem 6). This in turn implies that all the trajectories of network (67) converge towards the same 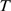-periodic solution. That is, all the nodes of (67) synchronize onto a periodic orbit of period 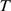.

### A counterexample to entrainment

**Figure 14:**
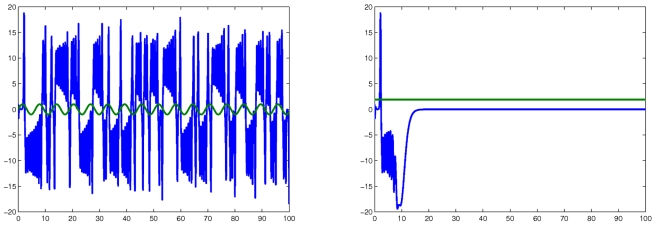
Simulation of counter-example. The following randomly-chosen input and initial conditions are used: 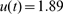, 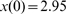 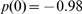, 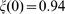, 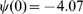, 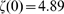. Green: inputs are 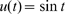 (left panel) and 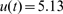 (randomly picked, right panel). Blue: 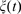. Note chaotic-like behavior in response to periodic input, but steady state in response to constant input.

In [5] there is given an example of a system with the following property: when the external signal 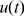 is constant, all solutions converge to a steady state; however, when 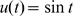, solutions become chaotic. (Obviously, this system is not contracting.) The equations are as follows:
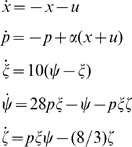
where 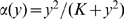 and 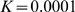. Figure 14 shows typical solutions of this system with a periodic and constant input respectively. The function “rand” was used in MATLAB to produce random values in the range 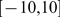.

## Discussion

We have presented a systematic methodology to derive conditions for various types of biochemical systems to be globally entrained to periodic inputs. For concreteness, we focused mainly on transcriptional systems, which constitute basic building blocks for more complex biochemical systems. However, the results that we obtained are of more generality. To illustrate this generality, and to emphasize the use of our techniques in synthetic biology design, we discussed as well the entrainment of a Repressilator circuit in a parameter regime in which endogenous oscillations to not occur, as well as the synchronization of a network of Repressilators. These latter examples serve to illustrate the power of the tools even when a large amount of feedback is present.

Our key tool is the use of contraction theory, which we believe should be recognized as an important component of the “toolkit” of systems biology. In all cases conditions are derived by proving that the module of interest is contracting under appropriate generic assumptions on its parameters. A surprising fact is that, for these applications, and contrary to many engineering applications, norms other than Euclidean, and associated matrix measures, must be considered. Of course, more than one norm may be appropriate for a given problem: for example we can pick different 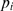
's in our weighted norms, and each such choice gives rise to a different estimate of convergence rates. This is entirely analogous to the use of Lyapunov functions in classical stability analysis: different Lyapunov functions provide different estimates.

Ultimately, and *as with any other method for the analysis of nonlinear systems*, such as the classical tool of Lyapunov functions, finding the “right” norm is more of an art than a science. A substantial amount of trial and error, intuition, and numerical experimentation may be needed in order to come up with an appropriate norm, and *experience with a set of already-studied systems* (such as the ones studied here) should prove invaluable in guiding the search.

## References

[1] Gonze D, Bernard S, Walterman C, Kramer A, Herzerl H (2005). Spontaneous synchronization of coupled circadian oscillators.. Biophysical Journal.

[2] Tyson JJ, Csikasz-Nagy A, Novak B (2002). The dynamics of cell cycle regulation.. Bioessays.

[3] Mettetal JT, Muzzey D, Gomez-Uribe C, van Oudenaarden A (2008). The frequency dependence of osmo-adaptation in Saccharomyces Cerevisiae.. Science.

[4] Kuznetsov YA (2004). Elements of applied bifurcation theory..

[5] Sontag ED (2009). An observation regarding systems which converge to steady states for all constant inputs, yet become chaotic with periodic inputs.. http://arxiv.org/abs/0906.2166.

[6] Lohmiller W, Slotine JJE (1998). On contraction analysis for non-linear systems.. Automatica.

[7] Lohmiller W, Slotine JJE (2000). Nonlinear process control using contraction theory.. AIChe Journal.

[8] Angeli D (2002). A Lyapunov approach to incremental stability properties.. IEEE Transactions on Automatic Control.

[9] Pham QC, Tabareau N, Slotine JJE (2009). A contraction theory approach to stochastic incremental stability..

[10] Granas A, Dugundji J (2003). Fixed Point Theory..

[11] Hartman P (1961). On stability in the large for systems of ordinary differential equations.. Canadian Journal of Mathematics.

[12] Lewis DC (1949). Metric properties of differential equations.. American Journal of Mathematics.

[13] Pavlov A, Pogromvsky A, van de Wouv N, Nijmeijer H (2004). Convergent dynamics, a tribute to Boris Pavlovich Demidovich.. Systems and Control Letters.

[14] Lohmiller W, Slotine JJ (2005). Contraction analysis of non-linear distributed systems.. International Journal of Control.

[15] Jouffroy J, Slotine JJE (2004). Methodological remarks on contraction theory..

[16] Slotine JJE, Wang W, Rifai KE (2004). Contraction analysis of synchronization of nonlinearly coupled oscillators..

[17] Wang W, Slotine JJE (2005). On partial contraction analysis for coupled nonlinear oscillators.. Biological Cybernetics.

[18] di Bernardo M, Russo G, Slotine JJ (2009). An algorithm to prove contraction, consensus and network synchronization..

[19] Russo G, di Bernardo M (2009). How to synchronize biological clocks.. Journal of Computational Biology.

[20] Russo G, di Bernardo M (2009). An algorithm for the construction of synthetic self synchronizing biological circuits..

[21] Sontag ED (1998). Mathematical Control Theory. Deterministic Finite-Dimensional Systems..

[22] Angeli D, Sontag ED (1999). Forward completeness, unboundedness observability, and their Lyapunov characterizations.. Systems and Control Letters.

[23] Michel AN, Liu D, Hou L (2007). Stability of Dynamical Systems: Continuous, Discontinuous, and Discrete Systems.

[24] Dahlquist G (1959). Stability and error bounds in the numerical integration of ordinary differential equations..

[25] Lozinskii SM (1959). Error estimate for numerical integration of ordinary differential equations.. I. Izv Vtssh Uchebn Zaved Matematika.

[26] Strom T (1975). On logarithmic norms.. SIAM Journal on Numerical Analysis.

[27] Arnold VI (1978). Mathematical methods of classical mechanics..

[28] Vidyasagar M (1993). Nonlinear systems analysis (2nd Ed.)..

[29] Del Vecchio D, Ninfa AJ, Sontag ED (2008). Modular cell biology: Retroactivity and insulation.. Nature Molecular Systems Biology.

[30] Slotine J (2003). Modular stability tools for distributed computation and control.. International Journal of Adaptive Control and Signal Processing.

[31] Elowitz MB, Leibler S (2000). A synthetic oscillatory network of transcriptional regulators.. Nature.

[32] Garcia-Ojalvo J, Elowitz MB, Strogatz SH (2004). Modeling a synthetic multicellular clock: Repressilators coupled by quorum sensing.. Proceedings of the National Academy of Science.

[33] Zhou T, Zhang J, Yuan Z, Xu A (2007). External stimuli mediate collective rhythms: artificial control strategies.. PLoS ONE.

[34] Wang R, Chen L, Aihara K (2006). Synchronizing a multicellular system by external input: an artificial control strategy.. Bioinformatics.

[35] Li C, Chen L, Aihara K (2007). Stochastic synchronization of genetic oscillator networks.. BMC Systems Biology.

